# Seven Cases of Zika Virus Infection in South Florida

**DOI:** 10.7759/cureus.1099

**Published:** 2017-03-16

**Authors:** Waqaar Khawar, Romina Bromberg, Molly Moor, Natalya Lyubynska, Hilda Mahmoudi

**Affiliations:** 1 Department of Internal Medicine, Aventura Hospital and Medical Center; 2 GME, Aventura Hospital and Medical Center

**Keywords:** zika virus, tropical disease, public health

## Abstract

**Introduction:**

Zika virus, an arbovirus of the *Flaviviridae* family, is a mosquito-borne virus known to cause microcephaly through vertical transmission. Infection presents with mild, self-limiting symptoms. Currently, a Zika virus outbreak has spread across most of South and Central America. Travel-related and sexually transmitted cases have been reported across the United States. However, the vector-borne transmission has been limited to Florida and Texas. We present seven cases of Zika virus infection that presented at a single institution in South Florida.

**Methods:**

Patients were included that had real-time polymerase-chain reaction (RT-PCR) for Zika virus RNA in urine or serum or enzyme-linked immunosorbent assay (ELISA) for Immunoglobulin M (IgM) antibody against Zika virus in serum.

**Results:**

All seven patients reported recent travel or employment in areas of active Zika virus transmission and at least two of the four most commonly reported symptoms (fever, arthralgia, rash, and conjunctivitis) with a rash present in all patients. All patients had positive RT-PCR for Zika virus RNA in urine. RT-PCR for Zika virus RNA in serum was negative in four of five patients that were tested, indicating that these patients likely presented one to two weeks after symptom onset.

**Conclusion:**

The future of Zika virus outbreaks in other cities in the United States is still uncertain. However, it is clear that prevention and control policies are urgently needed. We have presented seven confirmed cases of Zika virus infection in South Florida. In addition to conducting research concerning both the diagnostic and therapeutic aspects of the virus, there is a need for public awareness of its presentation, methods of transmission, and subsequent clinical outcomes.

## Introduction

Zika Virus (ZIKV) is an arbovirus of the Flaviviridae family that is most commonly transmitted via Aedes aegypti and Aedes albopictus mosquito vectors [1]. Other mechanisms of transmission include blood transfusion, sexual transmission, transplacental transmission, and laboratory exposure [2].

In 1947, ZIKV was first discovered in rhesus monkeys in the Zika Forest of Uganda. The first human cases were reported in Nigeria in 1954. ZIKV spread to Malaysia by 1969 and Indonesia by 1977. The first major ZIKV outbreak spread by mosquitos was recognized in 2007 in the Yap Islands of Micronesia, followed by a second larger outbreak in French Polynesia between 2013-2014 [3]. More recently, ZIKV has been reported in the Western Hemisphere, first in Chile and Brazil, followed by several other South and Latin American countries [4].

Due to its association with a rapid, 20-fold increase in the incidence of microcephaly in Brazil [5], concern regarding ZIKV increased significantly. As a result of the devastating adverse effects of ZIKV infection in utero, the World Health Organization (WHO) declared ZIKV a public health emergency of international concern [6].

Travel-related cases began appearing in the United States in 2015 [7]. In January 2016, the United States reported mosquito-borne transmission in Florida [7-8]. In addition, Texas was the first state where sexually transmitted ZIKV infection was reported [9]. A small number of locally transmitted cases have been reported in Texas as well [10]. By August 2016, there were 42 confirmed cases of ZIKV local transmission via mosquitos reported in Miami, Florida [11]. In 2016, there were 1,325 reported cases of ZIKV infection in Florida. Of these, 1,042 were travel-related, 262 were local transmissions, and 224 were in pregnant women. As of November 22, 2016, South Florida had no reports of locally transmitted ZIKV in 45 days though travel-related cases have continued to appear. As of January 27, 2017, there have been four cases in Florida; all of which were travel related [12]. The elimination of local transmission followed governmental efforts to decrease transmission via vector control and occurred in winter when the Aedes aegypti populations are less than 20% of their peak annual abundance in South Florida. In fact, historical population data shows Aedes aegypti populations increased up to 10-fold in summer as compared to winter [13].

The incubation period between exposure and the onset of clinical manifestations is two to 13 days [14]. About 80% of infected individuals are asymptomatic. However, if symptomatic, those infected with ZIKV commonly present with fever, maculopapular rash, arthralgia, and conjunctivitis [14-15]. Other symptoms include headache, retro-orbital pain, myalgia, iridocyclitis, vertigo, axillary and inguinal lymphadenopathy, and gastrointestinal dysfunction. The illness is usually mild and symptoms resolve within approximately seven days [3]. For symptomatic and asymptomatic patient within two weeks of infection, real-time polymerase chain reaction (RT-PCR) is done on matched urine and serum samples. A negative RT-PCR does not exclude the virus and serum should be analyzed by enzyme-linked immunosorbent assay (ELISA) for Immunoglobulin M (IgM) antibody. ELISA for IgM is also indicated for patients infected for more than two weeks. Presumed positive or equivocal IgM tests should be confirmed by plaque-reduction neutralizing testing [16-17].

We present a case series of seven symptomatic patients with ZIKV who presented for evaluation.

## Materials and methods

Patients presenting with a history of exposure to Zika virus and common symptoms (e.g., fever, arthralgia, conjunctivitis, or maculopapular rash [14-15]) had blood and/or urine collected for testing. Exposure was defined as travel or residence in an area with active ZIKV transmission or sexual contact with somebody with exposure. All patients were included retrospectively that presented to our hospital and had positive RT-PCR for ZIKV RNA in serum or urine or positive ELISA for IgM antibody against ZIKV in serum. This study received IRB approval.

## Results

### Case 1

A 56-year-old male presented to the emergency department complaining of fever, joint pain, and a generalized rash for three days. He denied headache or ocular complaints. He denied any recent travel. The patient works outdoors at a hotel in the South Beach neighborhood of Miami Beach, Florida, an area known to have active vector-borne transmission of ZIKV at the time of the patient’s presentation [18]. On physical exam, the patient was afebrile and hemodynamically stable. He had a generalized erythematous maculopapular rash and no other relevant physical findings, including ocular manifestations. Blood urea nitrogen (BUN) and creatinine were elevated (BUN: 23 mg/dL, normal range: 6–22 mg/dL; creatinine: 1.32 mg/dL, normal range: 0.43–1.13 mg/dL). Otherwise, blood chemistry tests were within normal limits. Hematologic tests were all within normal limits. RT-PCR for ZIKV RNA was positive in urine, though the results were not available until after discharge, as the sample was analyzed at an offsite laboratory. Serum was not tested for ZIKV, chikungunya, or dengue viruses. The patient was treated with one dose of ketorolac 30 mg IV. On discharge, the patient was instructed to follow up with his primary care physician.

### Case 2

A 28-year-old female presented to the emergency department complaining of fever, generalized rash, arthralgia, and myalgia. She denied any headache or ocular complaints. She recently traveled to the Dominican Republic where she described having been bitten by mosquitos. History regarding trip duration and timing relative to symptom onset is not available. On physical exam, the patient was afebrile and hemodynamically stable. She had a generalized maculopapular rash and no other relevant physical exam findings, including ocular manifestations. Blood chemistry tests were all within normal limits. Hematologic tests showed very mildly increased monocyte percentage (10.8%, normal range: 0.0–10.0%) with a normal white blood cell count. Laboratory findings were positive for RT-PCR for ZIKV RNA in urine and negative in serum. Chikungunya (IgM and Immunoglobulin G [IgG]) and dengue (IgM) virus serology were negative. Body fluid samples were analyzed at an offsite laboratory; therefore, the results were not available until after discharge. A pregnancy test was negative. The patient was treated with one dose of each of the following medications: morphine 2 mg IV, ondansetron 4 mg IV, and methylprednisolone 80 mg IV. She was also given 1 L of normal saline IV. On discharge, the patient was instructed to follow up with her primary care physician.

### Case 3

A 33-year old female presented to the emergency department complaining of a gradually worsening rash localized to her chest, neck, and right side of her face for two days. Associated symptoms were fatigue, sore throat, nasal congestion, “itchy, red, watery eyes,” and myalgia. She denied fever, chills, and arthralgia. She had returned to the United States from the Dominican Republic one week prior, but she did not recall being bitten by a mosquito. She reported that her son had a positive test for ZIKV. On physical exam, the patient was afebrile and hemodynamically stable. She had an erythematous posterior pharynx and a trace fine papular rash on the chest, neck, and right face. Blood chemistry tests were all within normal limits. Hematologic tests showed mild anemia (hemoglobin 11.9 g/dL, normal range: 12.0–16.0 g/dL), mildly increased lymphocyte percentage (44.0%, normal range: 23.0–43.0%), and mildly increased monocyte percentage (10.7%, normal range: 0.0–10.0%). RT-PCR for ZIKV RNA was positive in urine and negative in serum. The patient was not tested for chikungunya or dengue virus infections. The ZIKV blood and urine samples were analyzed at an offsite laboratory; therefore, the results were not available at the time of discharge. A pregnancy test was negative. The patient was treated with one dose of each of the following medications: diphenhydramine 25 mg IV, acetaminophen 975 mg IV, and ondansetron 4 mg IV. She was also given 1 L of normal saline IV. On discharge, the patient was instructed to follow up with her primary care physician.

### Case 4

A 17-year-old male presented to the emergency department due to his father’s concern that the patient was infected with ZIKV. He had no complaints or symptoms at the time of the visit. The patient reported experiencing joint pain, pruritic generalized rash, headache, and fever on the previous day. He denied any ocular complaints. He had returned from Puerto Rico on the previous day. While in Puerto Rico, he was in contact with a friend who experienced the same symptoms. Per the patient, the friend’s physician told him he had a viral condition. On physical exam, he was afebrile and hemodynamically stable. There were no positive relevant findings on physical exam. Blood chemistry tests were all within normal limits. Hematologic tests showed a mildly decreased white blood cell count (3.1 × 10^3^/mL, normal range: 4.5–13.5 × 10^3^/mL) and a mildly increased hemoglobin (15.3 g/dL, normal range: 11.0–15.0 g/dL) and hematocrit (43.7%, 33.0–43.0%). Lymphocyte percentage was decreased (0.9 × 10^3^/µL, normal range: 1.2–6.4 × 10^3^/µL). RT-PCR for ZIKV RNA was positive in urine and negative in serum. Serology was positive for dengue virus (IgM positive, IgG negative) and negative for chikungunya virus (IgM and IgG negative). Body fluid samples were analyzed at an offsite laboratory; therefore, the results were not available at the time of discharge. The patient received no treatment during this visit. On discharge, the patient was instructed to follow up with his primary care physician and to avoid sexual contact.

### Case 5

A 56-year-old female presented to her primary care physician with fever, conjunctivitis, arthralgia, and a rash on her chest, arms, and neck. She reported recent travel to Jamaica. History regarding trip duration and timing relative to symptom onset is not available. Body fluid samples were collected, and RT-PCR for ZIKV RNA was positive in urine and negative in serum. Serum RT-PCR RNA for dengue virus was negative. Serology for dengue virus was negative for IgM and positive for IgG. Serum RT-PCR for chikungunya virus RNA was negative.

### Case 6

A 48-year-old male presented to his primary care physician with fever, pruritic rash, conjunctivitis, and joint pain for four days. Three days before the onset of symptoms, he returned with his wife (see Case 7 below) from a 12-day trip to Saint Martin and Saint Barthélemy: territories with known vector-borne ZIKV transmission [19]. The patient denied sexual contact with anybody who had “recently returned from a country where Zika has been spreading.” RT-PCR for ZIKV RNA in serum and urine as well as Zika IgM ELISA in serum were all positive. The patient was not tested for dengue or chikungunya virus.

### Case 7

A 49-year-old female presented to her primary care physician with fever and pruritic rash for four days. She denied symptoms of conjunctivitis and joint pain. Three days before the onset of symptoms, she returned with her husband (see Case 6 above) from a 12-day trip to Saint Martin and Saint Barthélemy: territories with known vector-borne ZIKV transmission [19]. The patient denied sexual contact with anybody who had “recently returned from a country where Zika has been spreading.” RT-PCR for ZIKV RNA was positive in urine. Serum was not tested for ZIKV (RNA or IgM) nor was serologic testing done for chikungunya or dengue viruses. ZIKV test results for all cases can be seen in Table 1.

**Table 1:** Laboratory results for Zika virus tests (+) = detected (-) = not detected ---- = not tested ELISA: Enzyme-linked immunosorbent assay; RT-PCR: Real-time polymerase chain reaction.

Patient	RT-PCR Serum	RT-PCR Urine	ELISA (IgM)
1	----	(+)	----
2	(-)	(+)	----
3	(-)	(+)	----
4	(-)	(+)	----
5	(-)	(+)	----
6	(+)	(+)	(+)
7	----	(+)	----

## Discussion

These seven cases illustrate the common clinical picture of a ZIKV infection. All patients had risk factors for acquiring the infection – whether by working in an active vector-borne area, person-to-person transmission, or traveling to endemic areas. The patients presented with classic signs and symptoms of ZIKV infection, including fever, chills, fatigue, maculopapular rash, arthralgia, headache, and conjunctivitis. Five patients (71%) had fever, arthralgia, and rash. All seven had rash, and at least one more of the common ZIKV infection symptoms. Only two patients (29%) had less than three of the common symptoms. One of these two patients presented with other less common symptoms (fatigue, myalgia, and pharyngitis). Six of the seven patients were symptomatic at the time of presentation, and all of their symptoms were self-limiting.

RT-PCR of ZIKV RNA was positive in urine for all seven cases. Four patients (57%) were negative for ZIKV RNA in serum (Table 1). These four patients’ presentations were all likely during the second week after onset of symptoms. RT-PCR of ZIKV RNA is unlikely to be detected in serum after seven days after onset of symptoms [16], whereas it remains detectable in urine up to at least 14 days [17]. Of the remaining three, one was positive for RT-PCR in serum and the others were not tested. Had their serum been tested, they likely would have been positive due to the fact that they presented three and four days after symptom onset (within the seven-day window) [16-17]. Serum IgM antibody against ZIKV and neutralizing antibodies detected by plaque reduction neutralization tests are detectable from four to seven days after onset of symptoms. These continue to be detectable in serum for years [17]. In serum testing in case 6, the patient was positive for both RT-PCR of ZIKV RNA and anti-Zika IgM by ELISA. These data indicate that the patient likely presented within four to seven days of symptom onset which is consistent with the patient’s history.

The patients in cases 6 and 7 were most likely infected while traveling, but local vector-borne infection cannot be ruled out. With the lower end of the range of the incubation period being two days after exposure [14], it is possible that the patients were infected by vector-borne transmission in South Florida within two days of their return from their trip.

Severe disease requiring hospitalization is uncommon, and case-fatality rates are low [20]. However, what is disconcerting is that the clinical sequelae of the virus have still not been fully investigated. ZIKV has been shown to be associated with Guillain-Barré [21-22], congenital microcephaly [5], and fetal losses among women infected during pregnancy [23]. Other neurologic complications have included myelitis [24], meningoencephalitis [25], brain ischemia, and death [26-28]. Although data regarding ZIKV infection on the molecular level is limited in humans, a study in adult mice showed ZIKV infection of neural progenitor cells, leading to reduced proliferation and cell death [29]. Due to the critical role of impaired neurogenesis in the development of Alzheimer’s disease [30], ZIKV may have implications in the development of Alzheimer’s and other memory disorders. Further studies at the molecular, clinical, and epidemiologic levels are necessary to assess the long-term consequences of ZIKV infection. 

## Conclusions

Although local transmission of ZIKV has not been reported in recent months, it remains a concern. As summer months arrive, Aedes aegypti populations have historically grown up to 10 times larger than in winter. Therefore, there is a need for public awareness of its presentation, methods of transmission, and subsequent clinical outcomes. Efforts in these areas now could serve to help prevent or limit local transmissions from returning to South Florida in the warmer months.
